# Supplementary material for: Persistent biotic interactions of a Gondwanan conifer from Cretaceous Patagonia to modern Malesia
Source: Commun Biol. 2020 Nov 25;3:708. doi: 10.1038/s42003-020-01428-9 (PMC7689466; doi:10.1038/s42003-020-01428-9)
Supplement: Supplementary file 1 — Supplementary Information [file 42003_2020_1428_MOESM1_ESM.pdf]

Supplementary Information for

Persistent biotic interactions of a Gondwanan conifer from Cretaceous Patagonia to modern Malesia

Michael P. Donovan, Peter Wilf, Ari Iglesias, N. Rubén Cúneo, and Conrad C. Labandeira

Michael Donovan
mdonovan@cmnh.org

Contents

Supplementary Note 1 Descriptions of fossil and extant insect and fungal damage on *Agathis*.
Supplementary Fig. 1 Elongate blotch mines (DT88) and serpentine mine associated with cf. *Agathis* sp. leaves from the Maastrichtian portion of the upper Lefipán Formation.
Supplementary Fig. 2 Blotch mines (DT88) associated with *Agathis immortalis* from the Danian Palacio de los Loros 2 locality.
Supplementary Fig. 3 Blotch mines (DT88) on *Agathis zamunerae* from the early Eocene Laguna del Hunco, Huitrera Formation.
Supplementary Fig. 4 Blotch mines (DT88) on *Agathis zamunerae* from early middle Eocene Río Pichileufú, La Huitrera Formation.
Supplementary Fig. 5 Blotch mines and putative leaf mines on modern *Agathis*.
Supplementary Fig. 6 Fungal damage on modern *Agathis*.
Supplementary Fig. 7 Serpentine mines on modern *Agathis australis*, *Agathis lanceolata*, *Agathis macrophylla*, *Agathis robusta*, *Agathis labillardierei*, *Agathis dammara*.
Supplementary Fig. 8 Serpentine mines on modern *Agathis borneensis*, *Agathis kinabuluensis*, *A. flavescens*.

Supplementary Note 1

Fossil blotch mines (DT88), descriptions of individual specimens

Blotch mines on cf. *Agathis* sp. from the Lefipán Formation, terminal Maastrichtian

MPEF-Pb 9839 (LefL; Fig. 1I and Supplementary Fig. 1A–C)

Near-complete, elongate blotch mine (Fig. 1I and Supplementary Fig. 1A). Preserved length is 49 mm and width is 5.0–8.3 mm. The mine is situated along the midline of the leaf with its long axis parallel to the veins. The width of the mine is greatest near the leaf base and gradually thins in the direction of the leaf apex. Mine lateral margins are smooth (Supplementary Fig. 1B), linear to gently rounded, and surrounded by a dark reaction rim 0.1–0.2 mm in width. The margin of the mine closest to the leaf apex has a semicircular terminus. Although the complete basal portion of the mine was not recovered, the converging basal mine margins appear to indicate a similar, semicircular shape basally as does the apical margins, suggesting that the mine is mostly complete. The mined area of the leaf is significantly lighter in preserved color than the surrounding unconsumed tissue, suggesting that the mine may have been a full-depth structure. The parallel leaf venation is distorted but faintly visible within the mine (Supplementary Fig. 1C), and frass is completely absent throughout.

MPEF-Pb 9826 (LefE; Supplementary Fig. 1D)

Elongate, thin blotch mine positioned along the leaf margin (Supplementary Fig. 1D, lower arrow). Mined tissue closer to the leaf apex appears to have been lost during preservation of the specimen. The preserved length of the mine is 15.7 mm, and the probable complete length is estimated as 31.0 mm. Mine width is 0.4–1.8 mm. Margins of the mine are smooth and surrounded by a dark reaction rim 0.2–0.4 mm in width. The margin of the mine closest to the leaf base is rectangular. No frass is preserved in the mine. Another possible blotch mine (Supplementary Fig. 1D, upper arrow) is located closer to the leaf apex, but most of the mined leaf tissue was not preserved. Alternatively, this damage could represent necrotic tissue caused by a fungal pathogen.

Blotch mines on *Agathis immortalis* from Palacio de los Loros 2, Salamanca Formation, early Danian

MPEF-Pb 5970 (Holotype of *Frondicuniculum flexuosum*, Fig. 1A–C and Supplementary Fig. 2A–C)

Elongate blotch mine along the leaf margin (Fig. 1A and Supplementary Fig. 1A). The mine is 25.2 mm long by 4.9–7.9 mm wide. Frass is composed of spheroidal pellets measuring 0.1 mm in diameter and surrounded by smaller fragments of amorphous frass that fills the mine. The frass is darker and more densely packed near the margin of the mine adjacent to the leaf margin. The texture within the mine is characterized by a subtly transversely oriented, bulbous surface. The margins of the mine are 0.2 mm wide and markedly undulous, characterized by multiple (2–3 or more) adjacent ridges of wrinkled tissue (Fig. 1C and Supplementary Fig. 2C). Leaf veins within the mine are faintly visible but distorted (Fig. 1C, Supplementary Fig. 2C).

MPEF-Pb 5960 (Paratype of *Frondicuniculum flexuosum*, Supplementary Fig. 2D, E)

Blotch mine that spans the width of the leaf (Supplementary Fig. 2D). The mine follows the right margin for 11.4 mm and the left margin for 13.3 mm. The width of the mine is 6.8–9.4 mm. The lateral mine margins are undulous, except where bounded by the leaf margin. The margins are characterized by a raised ridge of reaction tissue with wrinkled edges (Supplementary Fig. 2E) measuring 0.4–0.8 mm wide. The inside of the mine has a surface texture of low-relief, curvilinear sulci that tend to be oriented more or less transverse to the leaf axis. However, details such as possible frass are difficult to determine because of poor preservation.

MPEF-Pb 6007 (Paratype of *Frondicuniculum flexuosum*, Supplementary Fig. 2F, G)

Probable elongate blotch mine at the base of a leaf (Supplementary Fig. 2F) measures 13.0 mm long by 1.2–1.9 mm wide. The mine is slightly depressed into the leaf. The margins of the mine are undulous and surrounded by a 0.2 mm wide reaction rim composed of black carbonized material. The interior of the mine has a texture of fine bulbous excrescences (Supplementary Fig. 2G), possibly representing frass. However, individual pellets are not visible within the frass. Alternatively, the texture may represent tissue fragments resulting from larval chewing damage.

MPEF-Pb 6001 (Paratype of *Frondicuniculum flexuosum*, Supplementary Fig. 2H–J)

Partially-preserved elongate blotch mine (Supplementary Fig. 2H–J). Preserved length is 35.2 mm, and preserved width varies between 1.4–4.7 mm. The mine follows the leaf margin and expands across the width of the mine. The inside of the mine is characterized by a series of arcuate, subparallel sulci and crests oriented transverse to the leaf axis, attributable to the presence of solid frass pellets. The mine is depressed into the leaf, and the undulous mine margins are raised relative to the mine.

MPEF-Pb 5984 (Supplementary Fig. 2K–M)
Teardrop-shaped blotch mine with smooth, rounded margins (Supplementary Fig. 2K). The initial phase of the mine is a small eminence (Supplementary Fig. 2M) measuring 6.7 mm long by 1.8 mm wide. The mine decreases in width to 0.7 mm wide before veering away from the leaf margin. The larger, terminal portion of the blotch measures 12.0 mm long by 7.7 mm wide and follows the leaf margin for 3.1 mm. The interior mined area of the mine is darker in color than the surrounding leaf matrix (Supplementary Fig. 2L, M), with possible particulate frass and amorphous matter spread throughout. A circular area of missing tissue (2.8 mm diameter), representing either tissue lost during preservation or possibly the missing epidermis of an exit hole, is located near the center of the terminal phase of the blotch mine (Supplementary Fig. 2K).

Blotch mines on *Agathis zamunerae* from Laguna del Hunco, Huitrera Formation, early Eocene

MPEF-Pb 6336 (Holotype of *Frondicuniculum lineacurvum*, quarry LH06, Fig. 1D–F, Supplementary Fig. 3A–E)
Elongate blotch mine measuring 43.8 mm long (preserved portion) by 2.2–8.2 mm wide (Fig. 1D, E, and Supplementary Fig. 3A, B). Frass is composed of spherical and hemispherical pellets measuring 0.04–0.12 mm in diameter and surrounded by dark, amorphous matter (Fig. 1F and Supplementary Fig. 3C, D). The frass pellets tend to be positioned near the mine margin, with smaller pellets positioned close to the edge, although pellets of different size are mixed together. The preserved color of the pellets varies between yellowish-amber to dark brown. Hemispherical pellets have a hollowed out center, which may be preservational (Supplementary Fig. 3D inset). Margins of the mine are smooth and linear along the long-axis and gently curved at the apical margin (Supplementary Fig. 3E), and are surrounded by a dark reaction rim measuring 0.2 mm

wide. When the leaf split during collection, portions of the blade counterpart remained intact on the mine as lighter colored areas, showing the external surface of the leaf mine (Supplementary Fig. 3E). Supplementary Fig. 3B and 3C are camera lucida drawings of specimen (A), with Supplementary Fig. 3C at three times the magnification of Supplementary Fig. 3B.

MPEF-Pb 3160 (Paratype of *Frondicuniculum lineacurvum*, quarry LH06, Supplementary Fig. 3F)

Small, elongate blotch mine positioned along the leaf margin. The mine measures 7.2 mm long by 2.0 mm wide. Lateral margins of the mine are smooth and linear along the long axis and gently rounded at the ends. The lateral margins are surrounded by dark, black to brown reaction tissue of width 0.1–0.3 mm. No visible frass is present in the mine. Leaf tissue is not preserved in some parts of the mine and appears to have flaked off during collection.

MPEF-Pb 6375 (quarry LH27, Supplementary Fig. 3G)

Blotch pattern, probably caused by a fungal pathogen instead of a leaf miner. The blotch measures 10.3 mm long by 6.8 mm wide and is bounded by the left leaf margin (6.4 mm long). The blotch is surrounded by thickened, carbonized reaction rims (0.4–0.6 mm wide), except for the portion along the leaf margin. The wide reaction rim is typical of some fungal blotches. Preserved tissue in the interior of the blotch is darker than undamaged tissue outside, possibly representing a necrosis. Dark, carbonized material is concentrated in portions of the blotch, particularly on the side closest to the base of the leaf. The margins of the blotch are ragged due to fragmentation of the remnant reaction rim and possibly the primary parallel venation of the leaf.

MPEF-Pb 6367 (quarry LH13, Supplementary Fig. 3H)

Elongate, thin mine, or alternatively, necrotic tissue, along the leaf margin. The complete leaf and blotch were not recovered, and parts of the inner tissue of the blotch were torn before preservation of the leaf. The torn portions are not associated with reaction rims, suggesting that the damage occurred after the surrounding leaf tissue was already dead. The preserved part of the blotch measures approximately 22 mm long and varies between 1–4 mm wide, with thickened scar tissue along its margins (0.1–0.2 mm wide). No definitive frass is visible within the blotch. A small area of similar tissue located 1 mm away from the elongate blotch possibly represents a

less mature or aborted mine, although it could also be a second patch of necrotic tissue. The damaged zone of the small area above is 2.5 mm wide and 1 mm deep and is missing leaf tissue.

MPEF-Pb 6325 (quarry AL01, Supplementary Fig. 3I)
Thin, elongate blotch on a poorly preserved leaf, possibly made by a leaf miner or fungus. The blotch measures 10 mm long by 1.0–1.4 mm wide. The long axis of the blotch is parallel to the leaf venation. Blotch margins are poorly preserved but appear smooth. The blotch is located about 0.6–0.7 mm distant from the leaf margin. Internal characteristics of the blotch are impossible to discern because of poor preservation.

MPEF-Pb 6352 (quarry LH13, Supplementary Fig. 3J)
Probable necrotic tissue positioned along the leaf margin. The basal margin of the blotch is linear. The lateral margin of the blotch gently curves as it thins towards the leaf margin apically. A dark, thick reaction rim surrounds the margins of the blotch and measures 0.1–0.3 mm in width. Leaf veins within the blotch are well preserved and possible epidermal cells are visible. The blotch lacks evidence of frass and may be related to fungal infection instead.

MPEF-Pb 6331 (quarry LH04, Supplementary Fig. 3K, L)
Oval blotch near the leaf base of unknown origin. The blotch measures 5.5 mm in length by 0.9–3.1 mm in width. The preserved color of the blotch portion of the leaf is white, black (carbonized), and tan. A thin black reaction rim (~0.1 mm wide) surrounds the blotch. On the counterpart, a layer of rock is broken off revealing darkened tissue. The presence of frass is unclear, although the area preserved as a tan color near the base is covered in small black dots, which could be related to fungal infection.

Blotch mines on *Agathis zamunerae* from Río Pichileufú, Huitrera Formation, middle Eocene

USNM 545226 (Paratype of *Frondicuniculum lineacurvum*) (Fig. 1G, H, and Supplementary Fig. 4A, B)
Elongate blotch mine occupying a major portion, perhaps 85%, of the leaf (Fig. 1G, H, and Supplementary Fig. 4A), including the entire width along one portion of the mine. The mine is

50 mm long by 2.2–10.0 mm wide and is narrowed only apically. The margins of the mine are smooth and mostly follow the leaf margins (Supplementary Fig. 4A, B). Circular pits (0.3–0.4 mm in diameter), possibly representing the former positions of scale insects, are scattered across the mine (Supplementary Fig. 4B).

USNM 545229 (Supplementary Fig. 4C)
Possible blotch mine along the leaf margin. The blotch measures 6.5 mm long by 3.7 mm wide and is filled with a white material, possibly secondary mineralization, that has a cuspate inner margin toward the leaf center away from the leaf edge. The white material could be mineralized silk, part of a pupal cocoon constructed by a moth. The mine margins are smooth and surrounded by a rim of carbonized reaction tissue measuring 0.3–0.5 mm wide.

USNM 545224 (Supplementary Fig. 4D)
Possible blotch mine located near the base of the leaf. The blotch is faintly preserved and ovate-elliptical in shape, measuring 6.8 mm in length by 5.5 mm in width, with the long axis parallel to leaf venation. The mine is slightly depressed into the leaf, and a rim of raised tissue surrounds the mine. The interior of the mine has is slightly less carbonized material than the rest of the leaf matrix. An ellipsoidal impression measuring 5.6 mm long by 1.0–1.3 mm wide is adjacent to or possibly extends from the apical portion of the mine.

Damage type 251 descriptions

Putative linear blotch mines with breached epidermal tissue (DT251; Fig. 2A and Supplementary Fig. 4E–H) have a similar appearance to slot feeding holes, although their smooth, gently curving margins and epidermal flaps suggest leaf mining. The mines measure 3.5–13.8 mm in length and 0.6–4.4 mm in width, with their long-axes parallel to leaf venation. Some mines that are flanked by flaps of epidermal tissue measure 0.3–1.3 mm wide (Fig. 2A). On some specimens, epidermal tissue is preserved across the widths of the mines (Supplementary Fig. 4H). Epidermal tissue was breached in other areas, possibly due to environmental factors, such as leaf abrasion or in-vivo weathering (Fig. 2A and Supplementary Fig. 4G, H). Some mines are surrounded by black reaction rims, which measures 0.3–0.4 mm wide (Fig. 2A and Supplementary Fig. 4G, H). Similar mines at Río Pichileufú measure 3.3–36 mm in length by

0.1–0.4 mm in width (Fig. 2B and Supplementary Fig. 4I, J). Flaps of unconsumed tissues along the rims of the mines measure 0.1–0.2 mm wide and are surrounded by depressed scar tissue measuring 0.2–0.3 mm wide.

DT251 on *Agathis zamunerae* from Laguna del Hunco, Huitrera Formation, early Eocene

MPEF-Pb 6361 (quarry LH13, Fig. 2A and Supplementary Fig. 4E)

Three linear blotch mines with breached epidermal tissue. The breached area of the mine closest to the leaf apex is 6.26 mm long by 0.75–1.22 mm wide and flanked with flaps of tissue (0.29–0.68 mm wide). Closer to the leaf base, two mines are connected to each other by a 0.75 mm wide strip of epidermal tissue. The breached area of the upper mine is 3.48 long by 0.56–0.96 mm wide and surrounded by a 0.38–1.26 mm wide flap of tissue. The mine below it is 6.97 mm long by 0.60 mm wide with a 0.38–0.54 mm wide flap of tissue. A patch of mined tissue below the breached area is 4.77 long by 1.60–1.87 mm wide.

MPEF-Pb 6303 (quarry LH06, Supplementary Fig. 4F–H)

Two leaves, each associated with a blotch mine, attached to a branch. The blotch on the bottom leaf is 13.8 mm long by 0.9–3.1 mm wide and surrounded by a 0.3–0.4 mm wide reaction rim. Some epidermal tissue remains intact. The thinnest portion of the mine may indicate the origination point, and increased widths may be related to larval growth. The mine on the upper leaf is 12.6 mm long by 1.4–4.4 mm wide and surrounded by a 0.4 mm wide reaction rim.

DT251 on *Agathis zamunerae* from Río Pichileufú, Huitrera Formation, middle Eocene

USNM 545227 (Fig. 2B and Supplementary Fig. 4I, J)

Two thin blotch mines with breached epidermal tissue on a leaf. The breached area of the top mine is 3.60 mm long by 0.09–0.39 mm wide and surrounded by flaps of tissue (0.15–0.22 mm wide). The mine is surrounded by a depressed rim (0.20–0.33 mm wide). The bottom mine is 3.26 mm long by 0.10–0.35 mm wide and surrounded by a 0.10–0.18 mm wide reaction rim.

Insect damage on extant *Agathis*

Previously documented associations

Detailed documentation in the literature of folivorous insects associated with extant *Agathis*, especially leaf mining and galling insects, is limited (Supplementary Data 1). *Agathis australis* occurs on the North Island of New Zealand[1]. Insects associated with *A. australis* are well-documented compared to other *Agathis* species and include a variety of woodboring beetles[2–8] and termites[2,9]; external foliage feeding beetles[2], moths[2,10], and phasmids[11]; seed-feeding orthopterans[2,12]; a diaspidid scale insect; and a leaf mining beetle[13] and moth species[14]. *Parectopa leucocyma* (Lepidoptera: Gracillariidae)[14] mines are typically characterized by an initial blotch phase transitioning into a serpentine trail. The mines then follow the leaf margin and end in a gall near or in the petiole.

Agathis lanceolata is an emergent tree in New Caledonian rainforests[1]. Two scale insects, *Ceroplastes rubens* Maskell (Coccidae)[15–18] and *Chrysomphalus aonidum* Linnaeus (Diaspididae)[16,18,19], have previously been documented in association with this species.

Agathis montana is restricted to small, high elevation and wet area of the Mt Panié Range in New Caledonia[1]. The first, confirmed pest species of *A. montana, Pactola kuscheli* Mazur (Curculionidae) was recently described as a woodborer[20]. Other groups of insects reared from *A. montana* twigs include mostly borer species of Coleoptera (Cerambycidae, Chrysomelidae, Curculionidae, Endomychidae, and Ciidae), but also piercing and sucking Thysanoptera (unknown family), Hemiptera (Pentatomidae), and Diptera (unknown family)[20].

Agathis moorei grows in rainforests of New Caledonia[1]. Previously documented associations include the diaspidid *Chrysomphalus aonidum* and a variety of beetles (Brentidae, Curculionidae, Zopheridae)[16].

Agathis ovata occurs in shrublands and forests in New Caledonia[1]. A scale insect, *Nipaecoccus aurilanatus* Maskell (Pseudococcidae)[21,22], is associated with this plant-host species. Houard described a circular to elliptical gall on the upper surface of a leaf from this species, which housed a central chamber surrounded by a ringed cavity and thickened upper surface[23,24]. *Chrysomphalus aonidum* (Hemiptera: Diaspididae) scales[18] are also associated with this species.

Agathis macrophylla is native to Fiji, Vanuatu, and the Solomon Islands and grows in lowland to lower montane rainforests[1]. Previously documented associations includes wood-boring termites[25] and beetles (Curculionidae, Anobiidae)[26], a pseudococcid, *Nipaecoccus aurilanatus*[21,22], and the seed predator *Agathiphaga vitiensis* Dumbleton (Lepidoptera: Agathiphagidae)[27], whose larvae develop in and feed on the seeds.

Agathis atropurpurea is a rare species that grows in lower montane rainforests in Queensland, Australia[1]. Previously documented insect associations include two species of nemonychid weevils reared from male pollen cones[28].

Agathis robusta has a disjunct distribution in Queensland, Australia and New Guinea[1]. Previously documented insect associations from Australia includes *Conifericoccus agathidis* Brimblecombe[29–31], *Nipaecoccus agathidis* Williams[22,32], *Agathiphaga queenslandensis* Dumbleton[27], and unidentified weevil and moth species reared from the cones and seeds[33], respectively. In New Guinea, two species of moths have been collected from cones, possibly a species of *Tarphyscelis* (Yponeumetidae) and a moth similar to *Proselena* (Tortricidae)[33].

Agathis labillardierei is an emergent tree in lowland to lower montane rainforests in New Guinea[1]. Two species of moths have been collected from cones, possibly a *Tarphyscelis* sp. (Yponeumetidae) and a moth similar to *Proselena* (Tortricidae), both which were also collected on *A. robusta* in New Guinea[33].

Agathis dammara occurs in eastern Malesia in lowland to upland tropical rainforests[1]. Previously documented insect associations include weevils (Curculionidae)[26], two unidentified beetle species collected from the seeds[34], and termites[35].

Agathis borneensis ranges from Borneo to Sumatra[1]. Previously documented associations include the termite, *Coptotermes curvignathus* Holmgren, also associated with the commercial rubber tree *Hevea brasiliensis* Müll.Arg., in Peninsular Malaysia[36].

Extant blotch mines observed in this study, comparable to DT88

Agathis moorei (New Caledonia) – Elongate, full-depth blotch mine with smooth, linear margins (Supplementary Fig. 5A). The mine measures 25.3 mm long by 1.0-3.3 mm wide. The portion of the mine nearest the leaf apex follows the leaf margin from 9.0 mm before curving slightly away. The mine is surrounded by a thin reaction rim (0.1 mm wide).

Agathis macrophylla (Fiji, Vanuatu, and the Solomon Islands) – Elongate ellipsoidal blotch mines occur along the leaf margins (Supplementary Fig. 5B) and measure 11.5–28.1 mm long by 3.8–7.7 mm wide with 0.2–0.4 mm wide reaction rims. The long axes of the mines are parallel to the leaf veins, and the mine margins are smooth.

Agathis atropurpurea (Queensland, Australia) (Fig. 1J) – We found a blotch mine on a field (litter) specimen of *A. atropurpurea* on Mount Bartle Frere in Queensland. The mine is full-depth and located along the leaf margin. The mine measures 33.9 mm long by 4.5–7.2 mm wide with the long axis following the leaf venation. The margins of the mines are smooth and gently curving.

Agathis microstachya (Queensland, Australia) (Fig. 1L, M) – Partially-preserved blotch mines on damaged leaves. Preserved portion of the mines measure 2.2–8.3 mm long by 8.3–10.0 mm wide. Margins of the mines are wavy. Frass pellets are clustered 0.3–0.8 mm from the mine margins and measure 0.1–0.2 mm in diameter (Fig. 1L).

Agathis dammara (eastern Malesia) – An upper surface blotch mine (Fig. Supplementary Fig. 5C) is elongate-ellipsoidal and positioned along the central axis of a leaf. The mine measures 15.8 mm in length by 1.2–3.6 mm in width with 0.1 mm wide reaction rims. The long axis of the mine is parallel to the leaf veins, and mine lateral margins are smooth.

Agathis borneensis (Borneo to Sumatra) – Elongate ellipsoidal blotch mines coursing through upper or lower surface tissue are found along the leaf edges (Fig. 1K and Supplementary Fig. 5D) of *A. borneensis* leaves. The mines measure 17.5–45.1 mm in length by 1.4–12.0 mm in width with reaction rims measuring 0.1–0.2 mm wide. The long axes of the mines are oriented parallel to leaf veins. Possible silk was found in one mine, suggesting a lepidopteran miner

(Supplementary Fig. 5D, E). Frass is deposited as spheroidal pellets measuring 0.3 mm in diameter (Supplementary Fig. 5F).

Putative mines observed in this study, comparable to DT251

Agathis moorei (New Caledonia) – Blotch mines are elongate-ellipsoidal, typically with smooth, linear, parallel margins and positioned with their long axes parallel to the leaf veins (Supplementary Fig. 5G–I). The blotch mines measure 10.0–26.9 mm in length and 1.0–3.7 mm in width, and are full depth, leaving only epidermal tissue intact on both sides of the leaf. In some cases, the epidermal tissue was destroyed, leaving a slot-like hole (Supplementary Fig. 5H). The blotch mines are typically positioned along the central axis of the leaf.

Agathis microstachya (Queensland, Australia) (Supplementary Fig. 5J–L) – The species is associated with elongate ellipsoidal blotch mines positioned along the central axes of the leaves. The long axes of the mines are parallel to the leaf veins. The mines have smooth margins, and the surfaces of the blotches exhibit slight relief relative to the rest of the leaf surface. On specimens where the epidermal tissue has been removed, rough-textured, hackly surface abrasions can be observed on inner tissue (Supplementary Fig. 5K).

Agathis robusta (Queensland, Australia and New Guinea) – Elongate ellipsoidal slots from Queensland, Australia (Supplementary Fig. 5M) are flanked with flaps of necrotic tissue. The slots measure 6.1–11.7 mm long by 0.7–1.7 mm wide. The tissue flaps measure 0.2–0.9 mm in width and are surrounded by a thin raised rim measuring 0.1 mm wide. Based on the morphology of the slots, including the size, shape, and presence of the epidermal tissue rim, it is possible that the slots are actually blotch mines with breached epidermal tissue, similar in structure damage found on *A. moorei* in New Caledonia. Elongate, ellipsoidal blotch mines are positioned along leaf margins or at the apices of leaves. The mines measure 19.9–35.2 mm long by 1.3–10.0 mm wide. The margins of the mines are smooth and are surrounded by reaction rims measuring 0.1 mm in width. The long axes of the mines are positioned parallel to major leaf venation.

Fungal blotches

Some fungal damage associated with *Agathis* leaves can appear superficially similar to blotch mines (Supplementary Fig. 6). However, the fungal blotches can differ from blotch mines in a number of respects, including the the presence of pycnidia, irregular, poorly defined margins, prominent, expansive reaction tissue and necrotic tissue, and no evidence of removed tissue between epidermal layers. At LH, five specimens of *A. zamunerae* are marked by blotches of possible necrotic tissue (Supplementary Fig. 3G–L), which may have been caused by pathogenic fungi. Their overall appearance is similar to fungal blotches on extant *Agathis* leaves, but they are missing pycnidia or spores. Although the possible fungal damage on *Agathis* leaf fossils cannot be assigned to taxonomic group, their presence suggests that pathogenic fungi may have utilized *Agathis* leaf tissue since at least the Eocene.

Extant serpentine mines observed in this study

We found serpentine mines on nine species of extant *Agathis*, mostly made by unknown insects[14]. The extant serpentine mines differ from the single serpentine mine associated with cf. *Agathis* sp. at LefE (Fig. 2D, E, and Supplementary Fig. 1E, F) in that none has the strong association with parallel leaf veins seen in the Cretaceous specimen.

Agathis australis (North Island of New Zealand) – *Parectopa leucocyma* (Lepidoptera: Gracillariidae)[14] mines are common on this species. The mines typically begin with a blotch phase and then transition into a serpentine trail. The serpentine phase of the mine follows the leaf margin and finally induces a gall near the petiole (Supplementary Fig. 7A, B). The larva overwinters in the gall and then pupates externally[14].

Agathis lanceolata (New Caledonian) – A single serpentine mine specimen (Supplementary Fig. 7C) begins near the center of a leaf, where it courses parallel to the leaf veins, then turns towards the leaf margin and finally wraps around towards the center of the leaf, ending in a subtly expanded termination. The mine is packed with frass and has smooth, lateral margins.

Agathis macrophylla (Fiji, Vanuatu, and the Solomon Islands) – Serpentine mines ending in ellipsoidal terminal chambers are present (Supplementary Fig. 7D), as well as zigzagging serpentine mines that end in a gall near the base of a leaf (Supplementary Fig. 7E, F). The

transition from serpentine mine to petiole gall is similar to *Parectopa leucocyma* mines on *Agathis australis* in New Zealand, but it lacks the blotch phases often made by *P. leucocyma* larvae[14].

Agathis robusta (Queensland, Australia and New Guinea) – Very robust, full depth serpentine mines are tightly winding and filled with frass (Supplementary Fig. 7G, H). The mines often overlap, creating a blotch-like appearance (Supplementary Fig. 7H). Other serpentine mines are characterized by a tightly winding, intestiniform trajectory, which dramatically increases in width into an oval blotch. (Supplementary Fig. 7I).

Agathis labillardierei (New Guinea) – Serpentine mines are moderately sinusoidal and display slight width increases over the course of the mines as well as smooth margins (Supplementary Fig. 7I).

Agathis dammara (eastern Malesia) – Serpentine mines are characterized by overlapping paths with central frass trails composed of spheroidal pellets (Supplementary Fig. 7J). Some serpentine mines lack an expanded terminal chamber (Supplementary Fig. 7K, O), while others end in a polylobate terminal chamber (Supplementary Fig. 7M) or are entirely a linear-elongate blotch (Supplementary Fig. 7L) or a small gall-like excrescence with an ovate exit hole (Supplementary Fig. 7N).

Agathis borneensis (Borneo to Sumatra) – Serpentine mines are moderately sinusoidal and sometimes transition into a blotch phase composed of overlapping linear paths in parallel (Supplementary Fig. 8A, B) or end in an ovate terminal chamber (Supplementary Fig. 8C).

Agathis kinabaluensis grows in Malaysian Borneo, on Mt. Kinabalu, Sabah, and Mt. Murud, Sarawak[1]. Serpentine mines have a tightly overlapping trajectory and are intermittently packed with frass (Supplementary Fig. 8D), although individual pellets are not visible.

Agathis flavescens only occurs on two mountains in Peninsular Malaysia[1]. Serpentine mines are packed with frass and characterized by gradually increasing width (Supplementary Fig. 8E),

smooth, linear margins, and an overlapping, zigzagging trajectory leading to a blotch-like appearance (Supplementary Fig. 8F).

Supplemental Fig. 1 Elongate blotch mines (DT88) and serpentine mine associated with cf. *Agathis* sp. leaves from the Maastrichtian portion of the upper Lefipán Formation, Patagonian Argentina. (A) Elongate ellipsoidal blotch mine with smooth margins, counterpart to specimen in Fig. 1I. (Maastrichtian, LefL, MPEF-Pb 9839). (B) Smooth, linear margin of the blotch mine in Fig. 1I. (C) Distorted parallel leaf veins in the mine in Fig. 1I. (D) Possible linear blotch mine along the leaf margin (LefE, MPEF-Pb 9826). (E) Linear serpentine mines following leaf venation. Arrow expands to (F) (DT139; MPEF-Pb 9836). (F) Detail of frass trail in (E).

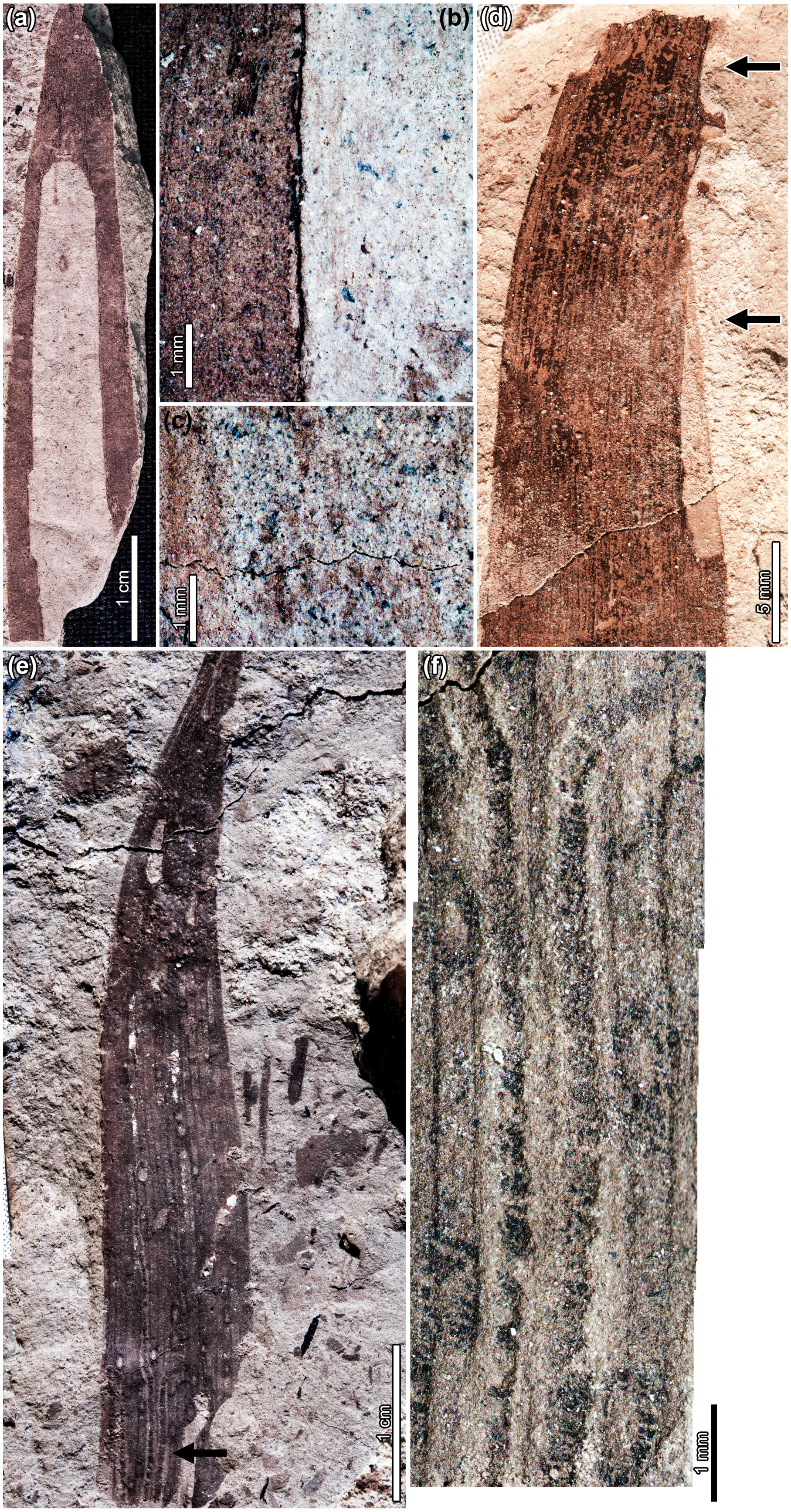
(a)
1 cm
(b)
1 mm
(c)
1 mm
(d)
5 mm
(e)
1 cm
(f)
1 mm

Supplementary Fig. 2 Blotch mines (DT88) associated with *Agathis immortalis* from the Palacio de los Loros 2 locality in the Danian Salamanca Formation, Patagonian Argentina. (A) Counterpart of the *Frondicuniculum flexuosum* specimen in Fig. 1A. Upper arrow expands to (C) and bottom arrow expands to (B) (holotype MPEF-Pb 5970). (B) Close-up of frass in (A). (C) Close-up of wrinkled, wavy margins and frass in (A). (D) Blotch mine spanning the width of the leaf. Arrow expands to (E) (MPEF-Pb 5960). (E) Close-up of wrinkled margin from (D). (F) Elongate blotch mine at the leaf base. Arrow expands to (G) (MPEF-Pb 6007). (G) Close-up of chewed tissue in blotch mine in (F). (H) Elongate blotch mine with wavy margins (MPEF-Pb 6001). (I) Close-up of elongate blotch mines in (H). (J) Counterpart of blotch mine in (I) and (J). (K) Blotch mine with smooth margins. Arrow expands to (M) (MPEF-Pb 5984). (L) Close-up of the inside of the terminal phase of the blotch mine in (K). (M) Close-up of initial phase of the blotch mine in (K).

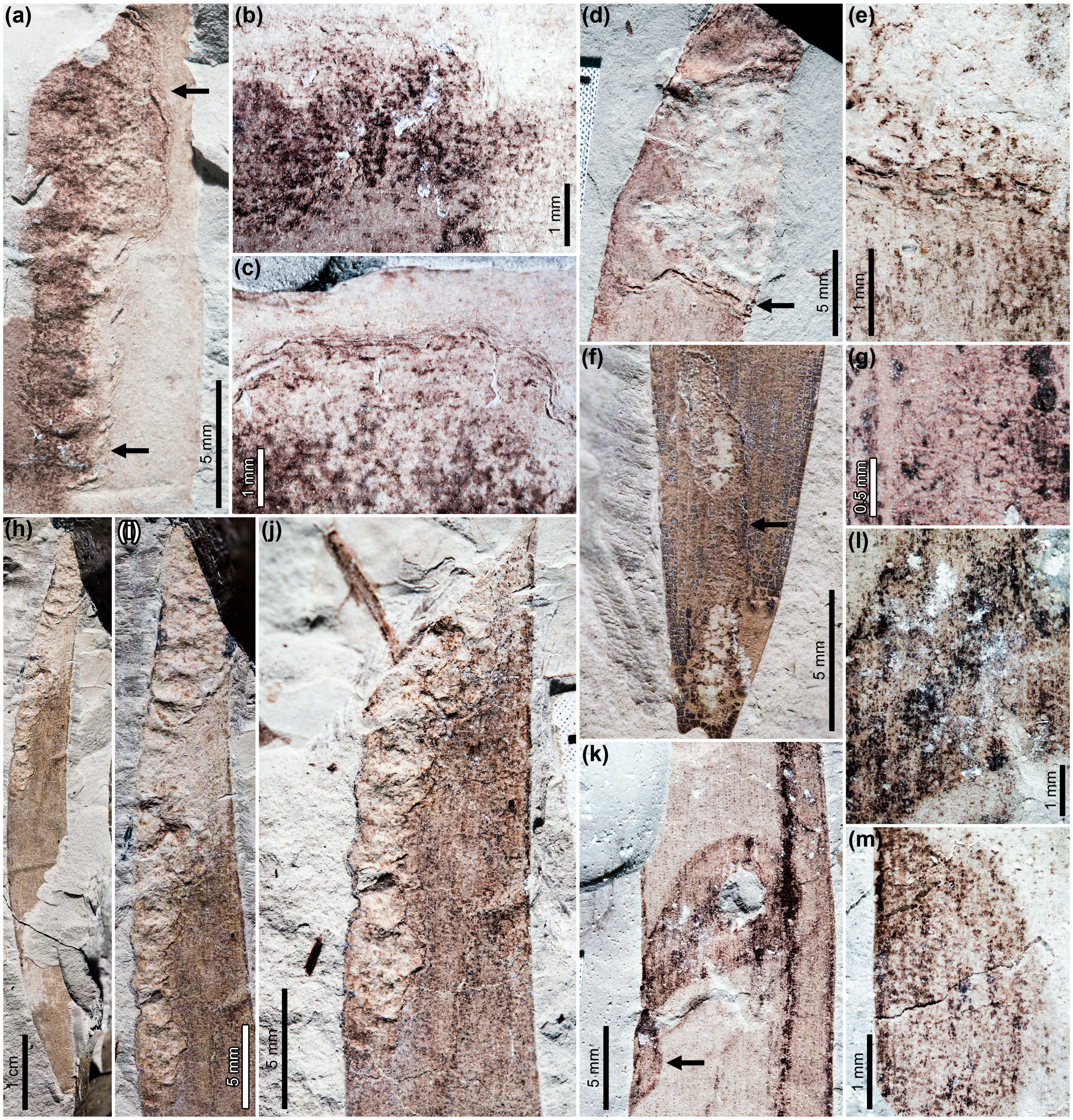
(a)
5 mm
(b)
1 mm
(c)
1 mm
(d)
5 mm
(e)
1 mm
(f)
5 mm
(g)
0.5 mm
(h)
cm
(i)
5 mm
(j)
5 mm
(k)
5 mm
(l)
1 mm
(m)
1 mm

Supplementary Fig. 3 Blotch mines (DT88) on *Agathis zamunerae* from the early Eocene Laguna del Hunco, Huitrera Formation in Patagonian Argentina. (A) Holotype of *Frondicuniculum lineacurvum*. Upper arrow expands to (D) and bottom arrow expands to (E) (holotype MPEF-Pb 6336 (B) Camera lucida drawing of (A). (C) Camera lucida drawing of frass in (A). (D) Close-up of frass along the mine margin (A), inset illustrates a hemispherical frass pellet with hollow center. (E) Close-up of smooth, gently curved mine margin from (A). (F) Blotch mine with smooth margins positioned along the leaf margin (MPEF-Pb 3160). (G) Possible fungal spot with thick reaction rim (MPEF-Pb 6375). (H) Elongate blotch mine or possible necrotic tissue caused by fungi along the leaf margin (MPEF-Pb 6367). (I) Possible poorly-preserved blotch mine or fungal damage (MPEF-Pb 6325). (J) Probable necrotic tissue along the leaf margin, possibly caused by fungi (MPEF-Pb 6352). (K) Possible fungal blotch or blotch mine at the leaf base (MPEF-Pb 6331). (L) Counterpart of possible blotch mine on K.

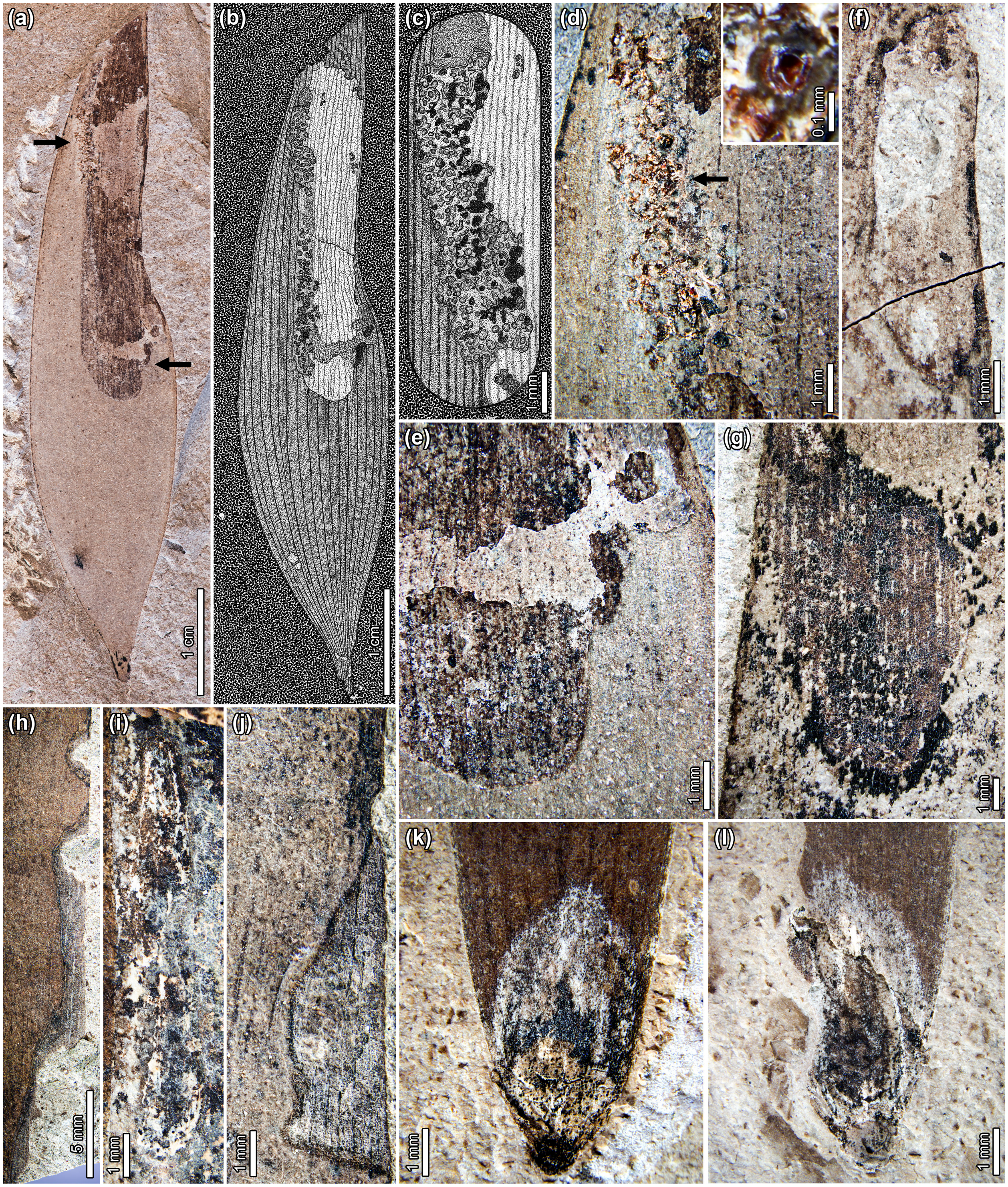
(a)
1 cm
(b)
1 cm
(c)
1 mm
(d)
0.1 mm
1 mm
(e)
1 mm
(f)
1 mm
(g)
1 mm
(h)
5 mm
(i)
1 mm
(j)
1 mm
(k)
1 mm
(l)
1 mm

Supplementary Fig. 4 Blotch mines (DT88) on *Agathis zamunerae* from early middle Eocene Río Pichileufú, La Huitrera Formation in Patagonian Argentina. (A) Close-up of blotch mine with smooth margins in Fig. 1G, H (USNM 545226). (B) Close-up of mine in Fig. 1G, H with pits where scale insect may have been positioned (USNM 545226). (C) Possible blotch mine along the leaf margin (USNM 545229). (D) Possible blotch mine at the base of the leaf (left side of photograph) (USNM 545224). (E) Blotch mines with breached epidermal tissue (DT12 and DT251; MPEF-Pb 6361). (F) Four leaves attached to a stem, two of which have probable blotch mines with breached epidermal tissue. Upper arrow expands to (H) and bottom arrow expands (G) (DT251; MPEF-Pb 6303). (G) Detail of blotch mine in (F). (H) Detail of blotch mine in (F). (I) Probable blotch mines with breached epidermal tissue. Arrow expands to (J) (DT251; USNM 545227). (J) Detail of blotch mine in (I).

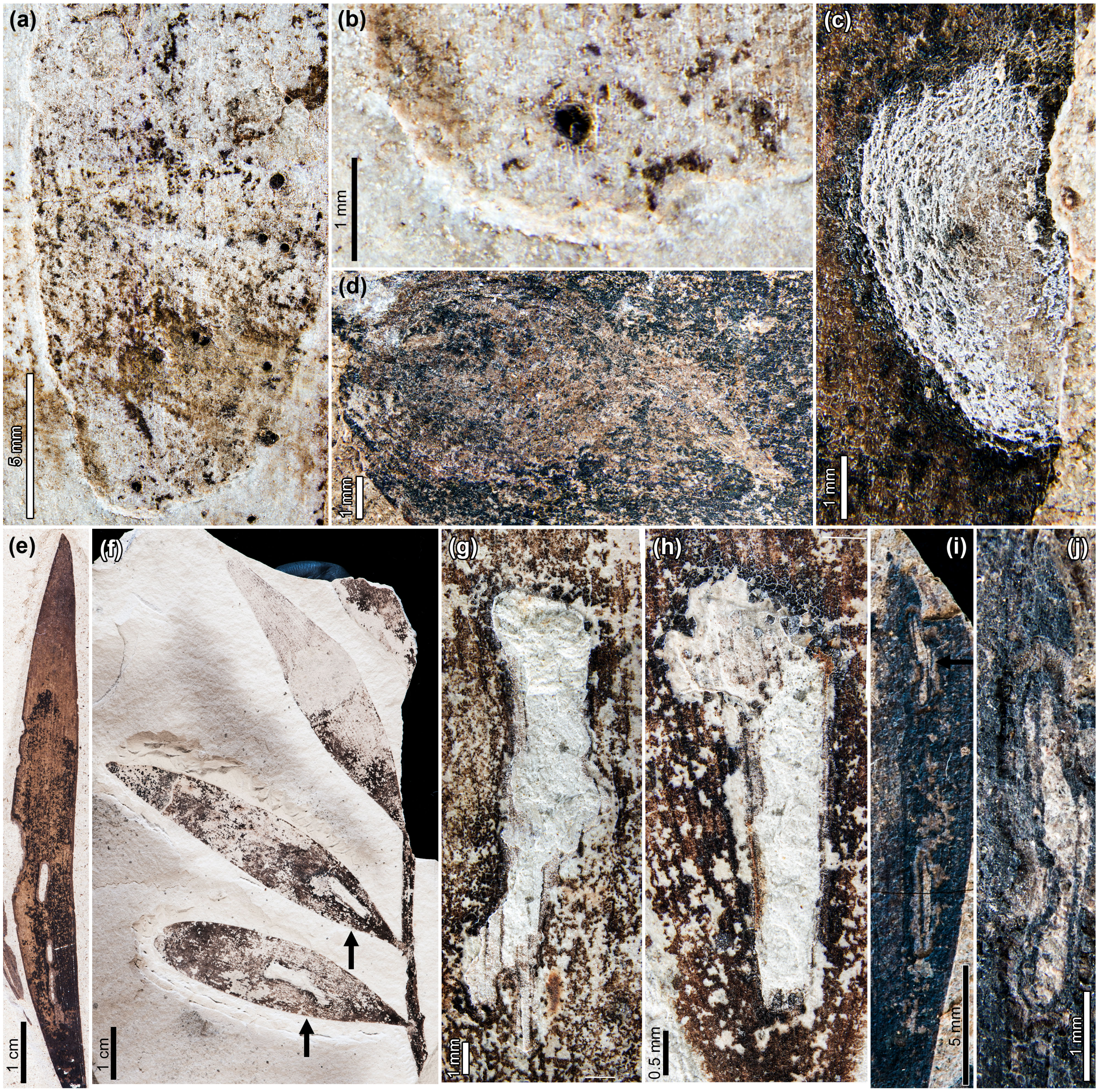
(a)
(b)
(c)
(d)
(e)
(f)
(g)
(h)
(i)
(j)
5 mm
1 mm
1 mm
1 mm
1 cm
1 cm
1 mm
0.5 mm
5 mm
1 mm

Supplementary Fig. 5 Blotch mines and putative leaf mines on modern *Agathis*. (A) Blotch mine with gently curving margins on *Agathis moorei* (New Caledonia, K 000553352). (B) Elongate blotch mine along the leaf margin on *Agathis macrophylla* (Fiji, K 0000327). (C) Ellipsoidal blotch mine along the central axis of the leaf (Maluku Islands, *D.S. Oey 121* (A)). (D) Elongate blotch mine with epidermal tissue removed exposing silk on *A. borneensis* (Brunei, K 000553179). (E) Detail of mine in D with possible silk (Brunei, K 000553179). (F) Detail of frass Fig. 1K (Brunei, SING 0091231). (G) Elongate ellipsoidal blotch mine along the central axis of an *A. moorei* leaf (New Caledonia, K 000553352). (H) Linear blotch mines with smooth margins on *Agathis moorei*. Epidermal tissue is breached on the right mine (New Caledonia, K 000553352). (I) Linear blotch mines on *Agathis moorei* (New Caledonia, K 000553352). (J) Ellipsoidal blotch mine with smooth margins on *Agathis microstachya* (Queensland, Australia, *B. Hyland 3303* (K)). (K) Blotch mine with epidermal tissue removed exposing surface abrasions from larval feeding on *Agathis microstachya* (Queensland, Australia, *B. Hyland 3303* (K)). (L) Ellipsoidal blotch mine with smooth margins on *Agathis microstachya* (Queensland, Australia, E 00210640). (M) Ellipsoidal, parallel-sided blotch mines. Epidermal tissue lost due to in-vivo weathering on *Agathis robusta* (Queensland, Australia, *T.S. Risley 82* (K)).

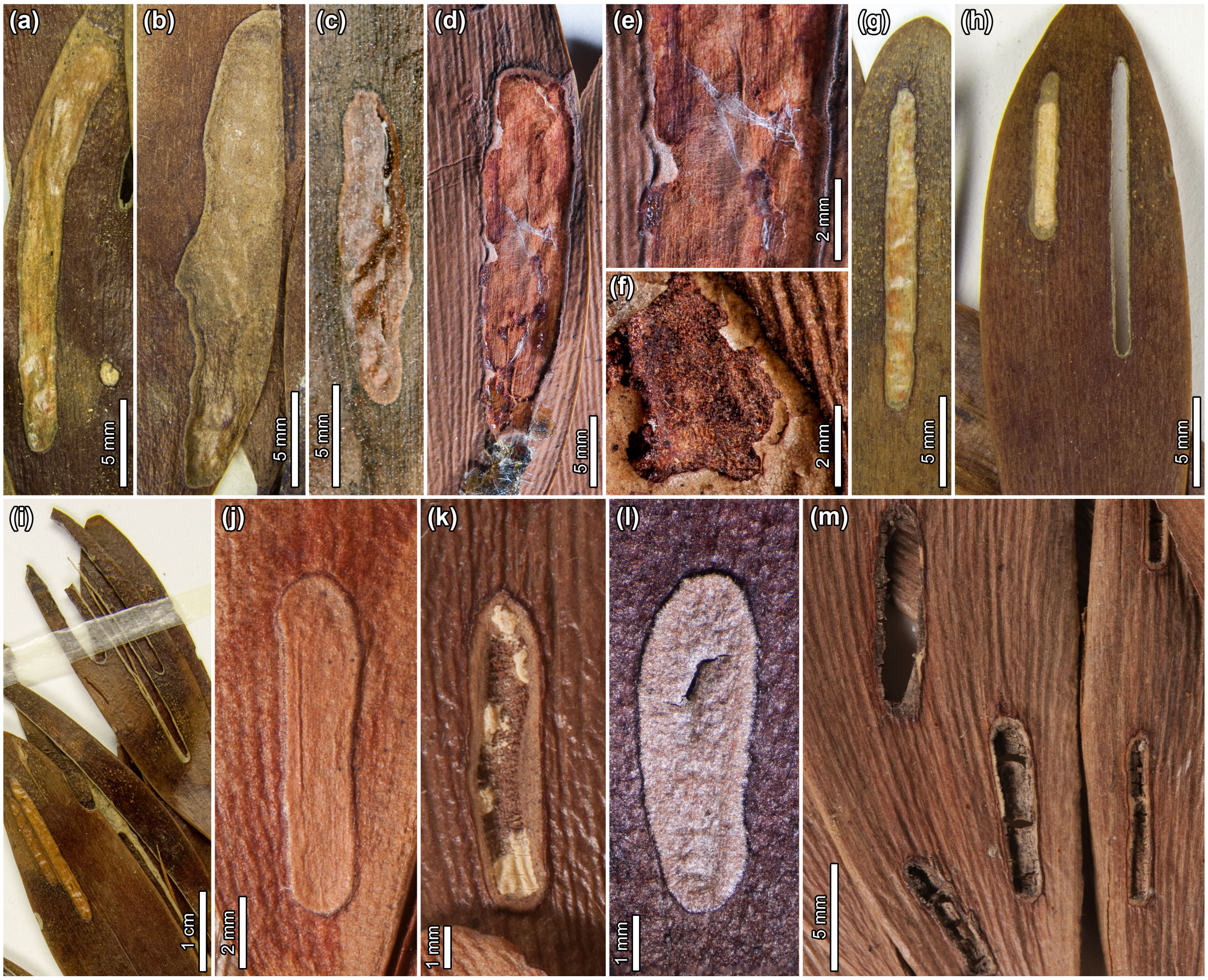
(a)
5 mm
(b)
5 mm
(c)
5 mm
(d)
5 mm
(e)
2 mm
(f)
2 mm
(g)
5 mm
(h)
5 mm
(i)
1 cm
(j)
2 mm
(k)
1 mm
(l)
1 mm
(m)
5 mm

Supplementary Fig. 6 Fungal damage on modern *Agathis*. (A) Field photograph of fungal blotch positioned along the leaf margin on *Agathis atropurpurea* leaf litter on Mount Bartle Frere, Queensland, Australia. (B) Close-up of opposite side of the fungal blotch shown in Supplementary Fig. 6A. (C) Necrotic tissue caused by fungi along the leaf margin of *Agathis robusta* (Queensland, Australia, A 01153261). (D) Fungal blotch along the leaf margin on *Agathis macrophylla* (Fiji, *D. Koroiveibau and J. Usumaki 16421*(K)).

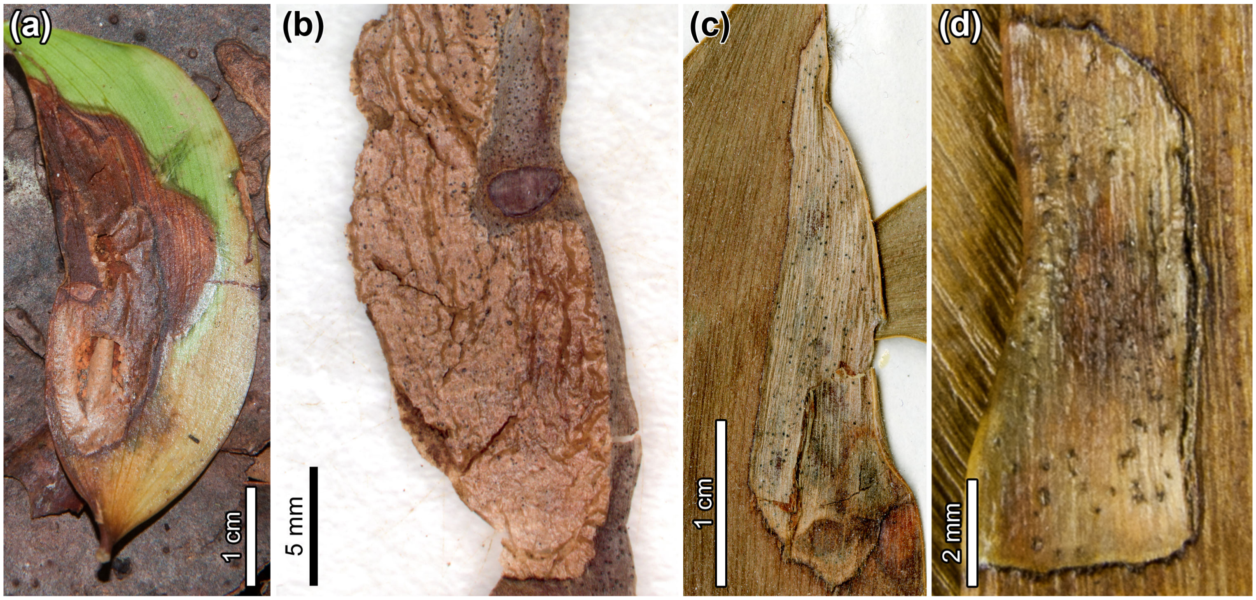

Supplementary Fig. 7 Serpentine mines on modern *Agathis*. (A) *Parectopa leucocyma* moth mine on *Agathis australis* (New Zealand, *Capt. Wilks, U.S.N., 1838-42* (GH)). (B) *Parectopa leucocyma* moth mines on *A. australis* (New Zealand, K 000553313). (C) Frass-filled serpentine mine on *Agathis lanceolata* (New Caledonia, K 000553127). (D) Serpentine mine ending in an ellipsoidal terminal chamber on *Agathis macrophylla* (Fiji, K 0000340). (E) Zigzagging serpentine mine ending in a gall near the petiole on *A. macrophylla* (Fiji, *D.J. de Laubenfels P502* (K)). (F) Zigzagging serpentine mine ending in a gall near the petiole on *A. macrophylla* (Fiji, *D.J. de Laubenfels P502* (K)). (G) Tightly winding serpentine mine on *Agathis robusta* (Queensland, Australia, CANB 590252). (H) Tightly winding serpentine mine ending in an ellipsoidal terminal chamber on *A. robusta* (Queensland, Australia, K 000553286). (I) Tightly sinusoidal serpentine mine on *Agathis labillardierei* (New Guinea, *Neth. Ind. For. Service bb. 30358* (SING)). (J) Overlapping serpentine mine with central frass trail on *Agathis dammara* (Maluku Islands, *D.S. Oey 121* (A)). (K) Serpentine mine on *A. dammara* (Maluku Islands, K 000553215). (L) An initial serpentine mine ending in an elongate blotch mine along the leaf margin of *A. dammara* (Maluku Islands, K 000553217). (M) Serpentine mine ending in a polylobate blotch on *A. dammara* (Sulawesi, Indonesia, *Neth. Ind. For. Service bb. 21570* (K)). (N) Serpentine mine ending in oval terminal chamber with exit hole on *A. dammara* (Sulawesi, Indonesia, *M.J.S. Sands 516* (K)). (O) Linear serpentine mine on *A. dammara* (Philippines, *T.C. Whitmore 3091* (K)).

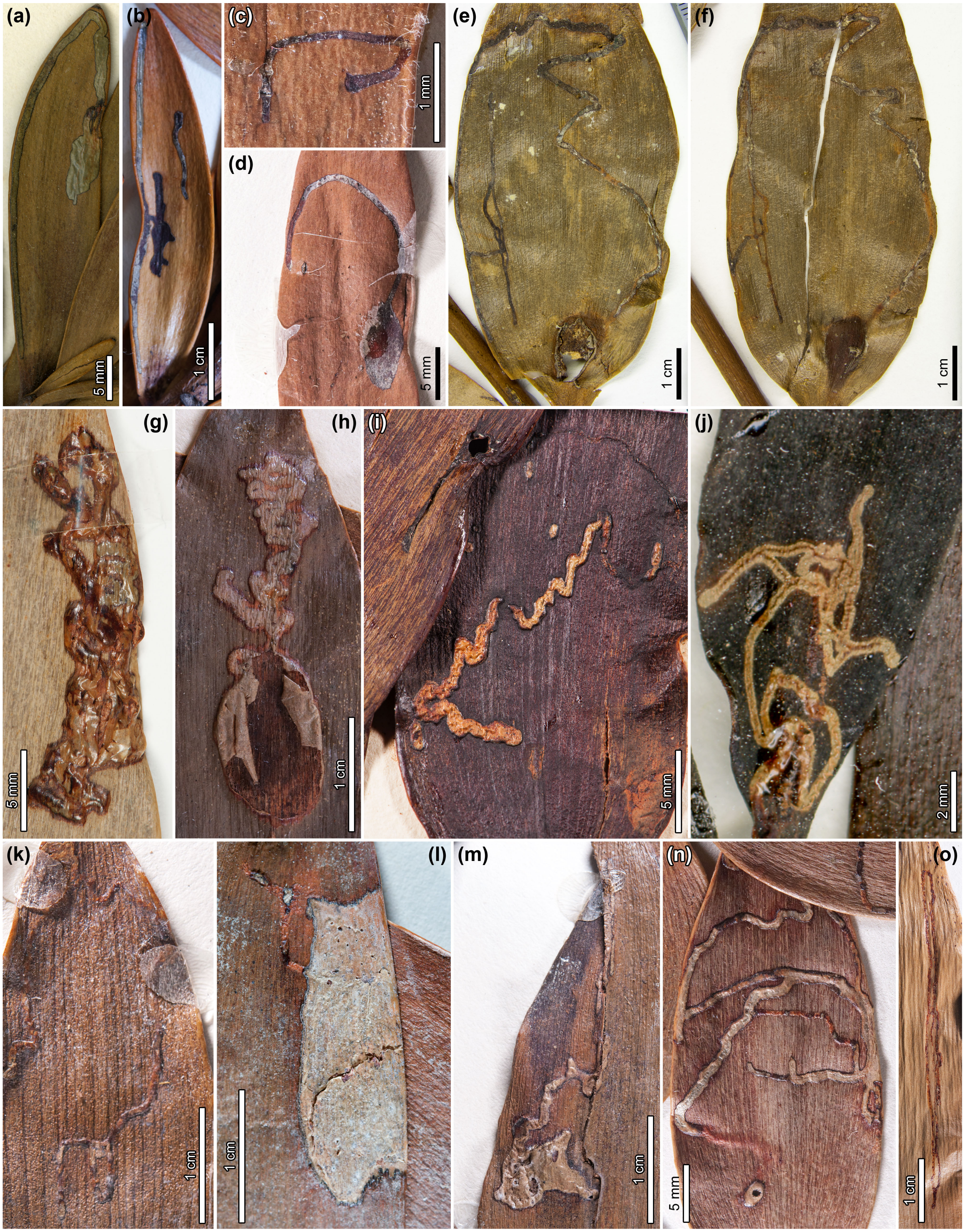
(a)
5 mm
(b)
1 cm
(c)
1 mm
(d)
5 mm
(e)
1 cm
(f)
1 cm
(g)
5 mm
(h)
1 cm
(i)
5 mm
(j)
2 mm
(k)
1 cm
(l)
1 cm
(m)
1 cm
(n)
5 mm
(o)
1 cm

Supplementary Fig. 8 Serpentine mines on modern *Agathis borneensis* (A-C), *Agathis kinabuluensis* (D), *A. flavescens* (E-F). (A) Frass-laden serpentine mine on *Agathis borneensis* (Penang Island, Malaysia, K 000553157). (B) Tightly sinusoidal serpentine mine on *A. borneensis* (Penang Island, Malaysia, SING 0090799). (C) Serpentine mine ending in an ovate terminal chamber on *A. borneensis* (Sulawesi, Indonesia, *H. Anang Atjil 4109* (A)). (D) Tightly overlapping serpentine mine on *Agathis kinabuluensis* (Sabah, Malaysia, *D.J. de Laubenfels P644* (K)). (E) Serpentine mines packed with frass on *Agathis flavescens* (Malaysia, *D.J. de Laubenfels P542* (A)). (F) Overlapping serpentine mine on *A. flavescens* (Malaysia, *D.J. de Laubenfels P542* (A)).

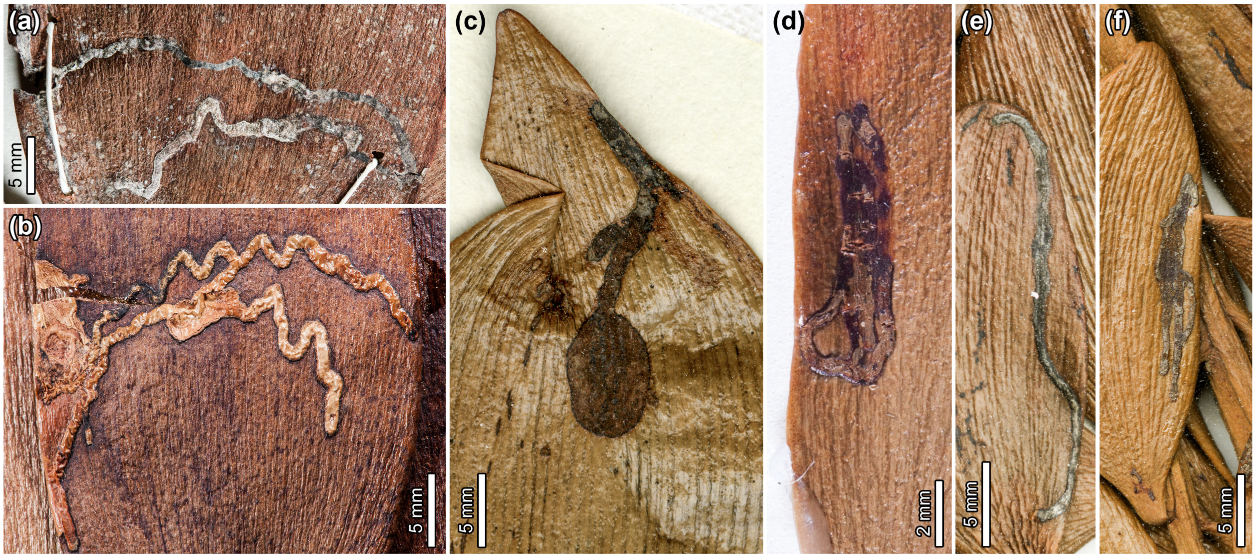

References

1. Farjon, A. *A Handbook of the World's Conifers*. (Brill, 2010).
2. Ecroyd, C. Biological flora of New Zealand 8. *Agathis australis* (D. Don) Lindl. (Araucariaceae) Kauri. *N. Z. J. Bot.* 20, 17–36 (1982).
3. Holloway, B. A. Anthribidae (Insecta: Coleoptera). *Fauna N. Z.* 3, 1–264 (1982).
4. May, B. M. Immature stages of Curculionoidea (Coleoptera): rearing records 1964-1986. *N. Z. Entomol.* 9, 44–56 (1987).
5. May, B. M. Larvae of Curculionoidea (Insecta: Coleoptera): a systematic overview. *Fauna N. Z.* 28, 1–226 (1993).
6. Kuschel, G. Nemonychidae, Belidae, Brentidae (Insecta: Coleoptera: Curculionoidea). *Fauna N. Z.* 45, 1–100 (2003).
7. Bain, J. *Pachycotes peregrinus* (Chapuis) (Coleoptera: Scolytidae). *For. Timber Insects N. Z.* 19, 1–4 (1977).
8. Lyal, C. C. H. Cryptorhynchinae (Insecta: Coleoptera: Curculionidae). *Fauna N. Z.* 29, 1–308 (1993).
9. Milligan, R. H. Insects damaging beech (*Nothofagus*) forests. *Proc. N. Z. Ecol. Soc.* 21, 32–40 (1974).
10. Nuttall, M. J. *Pseudocoremia fenerata* (Felder) (Lepidoptera: Geometridae) A native looper. *For. Timber Insects N. Z.* 56, 1–4.
11. Plant-SyNZ database. *http://plant-synz.landcareresearch.co.nz/* (2017).
12. Mirams, R. V. Aspects of the natural regeneration of the kauri (*Agathis australis* Salisb.). *Trans. R. Soc. N. Z.* 84, 661–680 (1957).

13. Martin, N. A. A longicorn leaf miner, *Microlamia pygmaea* (Coleoptera: Cerambycidae: Lamiinae) found in New Zealand. *N. Z. Entomol.* 23, 86 (2000).

14. Wise, K. A. J. *Parectopa leucocyma* (Meyrick) (Lepidoptera: Gracillariidae) rediscovered as a leaf-miner of kauri (*Agathis australis* Salisb.). *Trans. R. Soc. N. Z.* 1, 373–375 (1962).

15. Cohic, F. Contribution à l'étude des cochenilles d'intérêt économique de Nouvelle-Calédonie et Dépendances. *Comm. Pac. Sud Nouméa Doc. Tech.* 116, 1–35 (1958).

16. Brun, L. O. & Chazeau, J. *Catalogue des ravageurs d'intérêt agricole de Nouvelle-Calédonie*. (ORSTOM, Centre de Nouméa, 1986).

17. Williams, D. J. & Watson, G. W. *The Scale Insects of the Tropical South Pacific Region, Part 3: The Soft Scales (Coccidae) and Other Families*. (CAB International Institute of Entomology, 1990).

18. Mille, C., Henderson, R. C., Cazères, S. & Jourdan, H. Checklist of the scale insects (Hemiptera: Sternorrhyncha: Coccomorpha) of New Caledonia. *Zoosystema* 38, 129–176 (2016).

19. Williams, D. J. & Watson, W. *The Scale Insects of the Tropical South Pacific Region, Part 1: The Armoured Scales (Diaspididae)*. (CAB International Institute of Entomology, 1988).

20. Mazur, M. A., Tron, F. & Mille, C. *Pactola kuscheli* sp. nov. (Coleoptera: Curculionidae), a potential cause for the decline of the threatened New Caledonian conifer, *Agathis montana* de Laubenfels, 1969 (Araucariaceae). *Austral Entomol.* 56, 268–276 (2017).

21. Cox, J. M. Pseudococcidae (Insecta: Hemiptera). *Fauna N. Z.* 11, 1–232 (1987).

22. Ben-Dov, Y. *A systematic catalogue of the mealybugs of the world (Insecta: Homoptera: Coccoidea: Pseudococcidae and Putoidae) with data on geographical distribution, host plants, biology and economic importance.* (Intercept Limited, 1994).

23. Houard, C. Les collections cécidologiques du laboratoire d'entomologie du Muséum d'Histoire Naturelle de Paris: galles de Nouvelle-Calédonie. *Marcellia* 14, 143–182 (1914).

24. Houard, C. Les Zoocécidies des Plantes d'Afrique, d'Asie et d'Océanie. 1, 1–498 (1922).

25. Gray, B. Forest insect problems in the South Pacific islands. *Commonw. For. Rev.* 53, 39–48 (1974).

26. Praciak, A. *The CABI encyclopedia of forest trees*. (CAB International, 2013).

27. Dumbleton, L. A new genus of seed-infesting micropterygid moths. *Pac. Sci.* 6, 17–29 (1952).

28. Kuschel, G. *Nemonychidae of Australia, New Guinea and New Caledonia*. vol. 1 (CSIRO Australia, 1994).

29. Brimblecombe, A. R. Studies of the Coccoidea. 11. New genera and species of Monophlebidae. *Qld. J. Agric. Sci.* 17, 183–193 (1960).

30. Brimblecombe, A. R. & Heather, N. W. Occurrence of the kauri coccid, *Conifericoccus agathidis* Brimblecombe (Homoptera: Monophlebidae) in Queensland. *Aust. J. Entomol.* 4, 83–85 (1965).

31. Heather, N. W. & Schaumberg, J. B. Plantation problems of kauri pine in South East Queensland. *Aust. For.* 30, 12–19 (1966).

32. Williams, D. J. *Australian mealybugs.* (British Museum (Natural History), 1985).

33. Bowen, M. R. & Whitmore, T. C. A second look at *Agathis*. *CFI Occas. Pap.* 13, 1–19 (1980).

34. Zethner, O., Jorgensen, J. & Husaeni, E. A. The current status of diseases and pests of forest tree seeds in South East Asia, especially diseases in Indonesia. in *Proceedings of the ISTA*

Tree Seed Pathology Meeting Opocno, Czech Republic (eds. Prochazkova, Z. & Sutherland, J. R.) 86–94 (International Seed Testing Association, 1997).

35. Kiman, Z. B. Termites associated with *Agathis dammara* (Lamb.) L.C. Rich. plantation in Java. *BIOTROP Spec. Publ.* 20, 55–74 (1983).

36. Sajap, A. S., Mohamad, R. & Samah, R. A. The effectiveness of three preservatives and the relative resistance of ten Malaysian hardwoods against the subterranean termite, *Coptotermes curvignathus* (Holm.). *Pertanika* 5, 219–223 (1982).
