# Supplementary material for: Persistent biotic interactions of a Gondwanan conifer from Cretaceous Patagonia to modern Malesia
Source: Commun Biol. 2020 Nov 25;3:708. doi: 10.1038/s42003-020-01428-9 (PMC7689466; doi:10.1038/s42003-020-01428-9)
Supplement: Supplementary file 3 — Description of Additional Supplementary Files [file 42003_2020_1428_MOESM3_ESM.pdf]

Description of Additional Supplementary Files

File Name: Supplementary Data 1
Description: Folivores associated with extant *Agathis.*

File Name: Supplementary Data 2
Description: Fossil insect and fungal damage type data.
