# Supplementary material for: Persistent biotic interactions of a Gondwanan conifer from Cretaceous Patagonia to modern Malesia
Source: Commun Biol. 2020 Nov 25;3:708. doi: 10.1038/s42003-020-01428-9 (PMC7689466; doi:10.1038/s42003-020-01428-9)
Supplement: Supplementary file 6 — Reporting Summary [file 42003_2020_1428_MOESM6_ESM.pdf]

nature research

Corresponding author(s): Michael P. Donovan

Last updated by author(s): Jul 11, 2020

Reporting Summary

Nature Research wishes to improve the reproducibility of the work that we publish. This form provides structure for consistency and transparency in reporting. For further information on Nature Research policies, see our Editorial Policies and the Editorial Policy Checklist.

Statistics

For all statistical analyses, confirm that the following items are present in the figure legend, table legend, main text, or Methods section.

n/a	Confirmed	
☐	☒	The exact sample size (n) for each experimental group/condition, given as a discrete number and unit of measurement
☒	☐	A statement on whether measurements were taken from distinct samples or whether the same sample was measured repeatedly
☒	☐	The statistical test(s) used AND whether they are one- or two-sided<br>*Only common tests should be described solely by name; describe more complex techniques in the Methods section.*
☒	☐	A description of all covariates tested
☒	☐	A description of any assumptions or corrections, such as tests of normality and adjustment for multiple comparisons
☒	☐	A full description of the statistical parameters including central tendency (e.g. means) or other basic estimates (e.g. regression coefficient) AND variation (e.g. standard deviation) or associated estimates of uncertainty (e.g. confidence intervals)
☒	☐	For null hypothesis testing, the test statistic (e.g. F, t, r) with confidence intervals, effect sizes, degrees of freedom and P value noted<br>*Give P values as exact values whenever suitable.*
☒	☐	For Bayesian analysis, information on the choice of priors and Markov chain Monte Carlo settings
☒	☐	For hierarchical and complex designs, identification of the appropriate level for tests and full reporting of outcomes
☒	☐	Estimates of effect sizes (e.g. Cohen's d, Pearson's r), indicating how they were calculated

Our web collection on statistics for biologists contains articles on many of the points above.

Software and code

Policy information about availability of computer code

Data collection	No software was used.
Data analysis	No software was used.

For manuscripts utilizing custom algorithms or software that are central to the research but not yet described in published literature, software must be made available to editors and reviewers. We strongly encourage code deposition in a community repository (e.g. GitHub). See the Nature Research guidelines for submitting code & software for further information.

Data

Policy information about availability of data

All manuscripts must include a data availability statement. This statement should provide the following information, where applicable:

- Accession codes, unique identifiers, or web links for publicly available datasets
- A list of figures that have associated raw data
- A description of any restrictions on data availability

The data collected during this study are included in the published article and supplementary files.

Field-specific reporting

Ecological, evolutionary & environmental sciences study design

All studies must disclose on these points even when the disclosure is negative.

Study description	We compared insect herbivory and fungal damage associated with fossils of the conifer Agathis (Araucariaceae) from the latest Cretaceous to Eocene of Patagonia, Argentina to extant Agathis from its modern range in Australasia and Southeast Asia.
Research sample	We compared insect feeding damage on Patagonian fossil Agathis leaves, branches, and cone scales from well-studied localities of the latest Cretaceous (cf. Agathis sp., Lefipán Formation, localities LefE, LefL and, LefW, 12 specimens), early Paleocene (A. immortalis, Salamanca Formation, locality PL2, 319 specimens), early Eocene (A. zamunerae, LH, 121 specimens), and middle Eocene (A. zamunerae, RP, 32 specimens) with damage on extant Agathis species. The Lefipán localities and LH are located in northwest Chubut, Argentina; PL2 is in the western San Jorge Basin near Sarmiento in southern Chubut; and RP is located in western Rȉo Negro, Argentina.
Sampling strategy	All known Agathis fossils from Patagonia (latest Cretaceous to middle Eocene fossils curated at Museo Paleontológico Egidio Feruglio (MPEF-Pb), Museo de Paleontológico de Bariloche (BAR), San Carlos de Bariloche, Rȉo Negro, and Smithsonian National Museum of Natural History (USNM)) were compared to the extant Agathis specimens at the Arnold Arboretum (A) and Gray Herbarium (GH) of the Harvard University Herbaria, Royal Botanic Garden Edinburgh (E), Royal Botanic Gardens Kew (K), United States National Herbarium (US), Australian National Herbarium (CANB), National Herbarium of New South Wales (NSW), and the Singapore Botanic Gardens Herbarium (SING). We also examined the digitized (data.rbge.org.uk/search/herbarium) collections of Afrocarpus, Podocarpus, and Sundacarpus (Podocarpaceae) and Araucaria (Araucariaceae) from E and physical collections of Nageia (Podocarpaceae) from E and K.
Data collection	Donovan, Labandeira, Wilf, and Iglesias assigned damage types using the Guide to Insect (and Other) Damage Types on Compressed Plant Fossils (Labandeira et al., 2007) and photographed specimens. Donovan described the fossils.
Timing and spatial scale	Damage type data were collected and analyzed in 2014-2019.
Data exclusions	No data were excluded.
Reproducibility	Data used in this study are provided in the manuscript and supplemental material. Specimens are reposited in public institutions where they can be accessed for reproducibility.
Randomization	Not relevant.
Blinding	Not relevant.

Did the study involve field work? ☒ Yes ☐ No

Field work, collection and transport

Field conditions	Not relevant.
Location	The Lefipán localities and Laguna del Hunco are located in northwest Chubut, Argentina. Palacio de los Loros 2 is in the western San Jorge Basin near Sarmiento in southern Chubut, Argentina. Río Pichileufú is located in western Rȉo Negro, Argentina.
Access & import/export	Fossil specimens collected by the authors from Argentina are curated in the designated repository of the province from which they were found. Fossils from Lefipan, Palacio de los Loros 2, and Laguna del Hunco are curated at Museo Paleontológico Egidio Feruglio in Chubut, and fossils from Río Pichileufú are curated at Museo de Paleontológico de Bariloche (BAR), San Carlos de Bariloche, Rȉo Negro.
Disturbance	No disturbances were caused by the study.

Reporting for specific materials, systems and methods

We require information from authors about some types of materials, experimental systems and methods used in many studies. Here, indicate whether each material, system or method listed is relevant to your study. If you are not sure if a list item applies to your research, read the appropriate section before selecting a response.

Materials & experimental systems

n/a	Involved in the study
☒	☐ Antibodies
☒	☐ Eukaryotic cell lines
☐	☒ Palaeontology and archaeology
☒	☐ Animals and other organisms
☒	☐ Human research participants
☒	☐ Clinical data
☒	☐ Dual use research of concern

Methods

n/a	Involved in the study
☒	☐ ChIP-seq
☒	☐ Flow cytometry
☒	☐ MRI-based neuroimaging

Palaeontology and Archaeology

Specimen provenance

The Lefipán localities and Laguna del Hunco are located in northwest Chubut, Argentina. Palacio de los Loros 2 is in the western San Jorge Basin near Sarmiento in southern Chubut, Argentina. Río Pichileufú is located in western Rìo Negro, Argentina. Permits were granted by the Secretaría de Cultura del Chubut and Secretaría de Cultura de Río Negro. Multiple permits were issued as needed for each field season from 1999 to present.

Specimen deposition

Museo Paleontológico Egidio Feruglio (MPEF-Pb), Trelew, Chubut Province, Argentina; Museo de Paleontológico de Bariloche (BAR), San Carlos de Bariloche, Rìo Negro, Argentina; Smithsonian National Museum of Natural History (USNM), Washington, DC, USA

Dating methods

NA

☐ Tick this box to confirm that the raw and calibrated dates are available in the paper or in Supplementary Information.

Ethics oversight

No ethical approval was required. Laws regarding fossil collecting in Argentina were followed, permits were secured, and the fossils were reposited in museums in Argentina.

Note that full information on the approval of the study protocol must also be provided in the manuscript.
